# MULTIMORBIDITY PATTERNS PREDICT THE ORDER OF PHYSICAL FRAILTY AND COGNITIVE IMPAIRMENT OCCURRENCE

**DOI:** 10.1093/geroni/igae098.1014

**Published:** 2024-12-31

**Authors:** Shuomin Wang, Qianyuan Li, Qian-Li Xue, Minhui Liu

**Affiliations:** Central South University, Changsha, Hunan, China (People’s Republic); Central South University, Changsha, Hunan, China (People’s Republic); Johns Hopkins University, Baltimore, Maryland, United States; Ningxia Medical University, Yinchuan, Ningxia, China

## Abstract

Prior studies showed the difference in temporal sequence of physical frailty and cognitive impairment occurrence. Chronic conditions often co-occur in specific disease patterns, that is multimorbidity patterns, revealing shared pathophysiological mechanisms and risk factors. However, whether different multimorbidity patterns predict the order of frailty and cognitive impairment occurrence remain unknown. Combined participants recruited in 2011 and 2015 from National Health and Aging Trends Study, a total of 6055 community-dwelling older adults free of frailty or cognitive impairment at baseline were followed for 4 years. Latent class analysis was conducted to identify multimorbidity patterns with clinical meaningfulness. We used Fine & Gray competing risks models to examine the associations between multimorbidity patterns and risk of different orders of frailty and cognitive impairment occurrence. Four multimorbidity patterns were identified: cardiometabolic, osteoarticular, cancer-dominated, and multisystem pattern. Compared to non-multimorbidity, all four multimorbidity patterns were associated with a higher risk of developing frailty first. Specifically, multisystem pattern had a threefold higher risk (Sub-distribution hazard ratios [SHR]= 3.07, 95% CI= 2.21-4.26). However, those in the cardiometabolic (SHR =0.99, 95% CI= 0.88-1.12), osteoarticular (SHR =0.96, 95% CI= 0.82-1.11), and cancer-dominated (SHR =0.93, 95% CI= 0.79-1.09) patterns had a decreased risk of cognitive impairment-first occurrence, even though not statistically significant. In addition, older adults only from multisystem pattern showed a higher risk of co-occurrence of frailty and cognitive impairment. Clinician should be aware of etiological heterogeneity between frailty and cognitive impairment, and thus provide more effective prevention strategies for those comorbid older adults.

